# Attention-Based Personalized Encoder-Decoder Model for Local Citation Recommendation

**DOI:** 10.1155/2019/1232581

**Published:** 2019-06-03

**Authors:** Libin Yang, Zeqing Zhang, Xiaoyan Cai, Tao Dai

**Affiliations:** ^1^School of Automation, Northwestern Polytechnical University, Xi'an, Shaanxi, China; ^2^School of Telecommunications Engineering, Xidian University, Xi'an, Shaanxi, China; ^3^School of Software Engineering, Xi'an Jiaotong University, Xi'an, Shaanxi, China

## Abstract

With a tremendous growth in the number of scientific papers, researchers have to spend too much time and struggle to find the appropriate papers they are looking for. Local citation recommendation that provides a list of references based on a text segment could alleviate the problem. Most existing local citation recommendation approaches concentrate on how to narrow the semantic difference between the scientific papers' and citation context's text content, completely neglecting other information. Inspired by the successful use of the encoder-decoder framework in machine translation, we develop an attention-based encoder-decoder (AED) model for local citation recommendation. The proposed AED model integrates venue information and author information in attention mechanism and learns relations between variable-length texts of the two text objects, i.e., citation contexts and scientific papers. Specifically, we first construct an encoder to represent a citation context as a vector in a low-dimensional space; after that, we construct an attention mechanism integrating venue information and author information and use RNN to construct a decoder, then we map the decoder's output into a softmax layer, and score the scientific papers. Finally, we select papers which have high scores and generate a recommended reference paper list. We conduct experiments on the DBLP and ACL Anthology Network (AAN) datasets, and the results illustrate that the performance of the proposed approach is better than the other three state-of-the-art approaches.

## 1. Introduction

The rapid development of science and technology leads to a substantial increase in the number of scientific papers. Researchers have to spend a lot of time and effort to find relevant papers. Many researchers use keyword search via scholar search engines, such as Google Scholar and Microsoft Academic Search, but there exist many instances where such a keyword query is either over broad, returning many papers that are loosely relevant to what the researcher really need, or too narrow, filtering many potentially relevant papers out or returning nothing at all [1–5]. To alleviate the above problems, many studies presented citation recommendation approaches which use a manuscript or a text segment instead of a set of keywords as query [6–8]. Citation recommendation approaches are categorized into two major types: local citation recommendation, which recommends relevant papers based on a citation context [9–11], and global citation recommendation, which recommends relevant papers based on a given manuscript [12–15]. This study focuses on local citation recommendation.

Most local citation recommendation approaches utilize scientific papers' and citation context's text content only [9, 16–18] and concentrate on how to narrow the semantic difference between the two text objects, completely neglecting other information. For instance, each author has his own word usage, grammar structure, writing style, and personal citation preference, while each venue has its own topic, and it only publishes papers related to that topic. However, such information which has been neglected by researchers may have direct influence on the local citation recommendation task's performance, helping researchers find more appropriate references for the given citation context and yielding better performance.

Recent studies show the encoder-decoder framework performs well in the field of machine translation [19–21] as it learns relations between pairs of variable-length text. In this paper, we deem the scientific papers and citation context as parallel pairs, integrate several information related to scientific papers in attention mechanism, and propose an attention-based encoder-decoder (AED) model to score scientific papers via the venue information, author information, and the given citation context information, in turn, to enhance the performance of local citation recommendation. In AED model, we first construct an encoder which utilizes TDNN [7] to represent a citation context as a vector in a low-dimensional space, then we construct an attention mechanism integrating venue information and author information, and apply RNN to construct a decoder; finally, we map the decoder's output into a softmax layer and get the score value of scientific papers. A recommended reference paper list is generated based on high-scoring scientific papers. To summarization, our major contributions are as follows:

1. An attention mechanism is introduced in the encoder-decoder framework; the attention mechanism combines author and venue information.
2. An attention-based encoder-decoder (AED) model is proposed for local citation recommendation.
3. Experimental results illustrate the efficiency of the proposed AED model.

We organize the rest of this paper as follows.  Section 2 reviews related work.  Section 3 constructs an attention-based encoder-decoder model.  Section 4 illustrates the proposed attention-based encoder-decoder model to solve the local citation recommendation task.  Section 5 illustrates the experimental results, and Section 6 concludes the paper.

## 2. Related Work

Local citation recommendation is to find an ordered list of scientific papers from a dataset, and these scientific papers can be acted as candidate citations for a given citation context [11, 22]. The citation context can be the citation sentence itself or the composition of several preceding sentences and succeeding sentences of the citation sentence [23]. Most of the existing works focus on considering content semantic relationship between the two text objects, i.e., citation context and scientific papers, and recommend relevant references.

He et al. [24] evaluated the relevance between a given citation context and a paper and proposed a local citation recommendation model. Furthermore, they developed to automatically analyze a manuscript that lacks a bibliography and to recognize candidate locations in the manuscript where citations are needed [25]. Duma et al. [26] used the function of Core Scientific Concepts to local citation recommendation. Tang et al. [27] developed a bilingual embedding model, learning latent semantics of citations and contexts. Peng et al. [28] explored utilizing both knowledge-based methods and word-embedding similarity measures to model the relatedness between the papers and the citation contexts. Zhou [29] constructed an ActiveCite system, which integrates collaborative filtering, content-based filtering, and citation analysis approaches. ActiveCite uses current citation sentence as the query sentence for local citation recommendation and extracts topic of the paper as query sentence for global citation recommendation. The advantages of ActiveCite are that it is more intelligent and can minimize the interruption time of the paper writing process. Meanwhile, the author conducted usability research from the user's point of view and put forward some suggestions on the constructions and optimization of citation recommendation system. Huang et al. [30] developed RefSeer which suggests candidate citations based on the input queries. RefSeer presents both topic-based global recommendation and citation-context based local recommendation.

As there exists vocabulary gap between the paper's content and the citation context's content, Lu et al. [31] proposed a translation retrieval model to bridge this gap. Huang et al. [32] proposed to use unique IDs to denote the cited papers; they regarded these papers as “word” in a novel language, and they used a translation model to estimate the translation probability of an ID based on the citing words. Furthermore, they built a neural network-based citation recommendation model by using the distributed representations of words and documents [10], but all the above approaches concentrate on citation contexts' content information and scientific papers' content information only, ignoring venue information and author information. Ebesu and Fang [11] solved the local citation recommendation problem by proposing a neural citation network, which utilized content information and author information, but ignoring venue information. In this paper, we deem scientific papers as candidate papers and introduce an attention mechanism incorporating venue information and author information, and then we propose a novel model which contains encoder, attention mechanism, and decoder to solve the local citation recommendation task.

## 3. Attention-Based Encoder-Decoder Model

**Figure 1:**
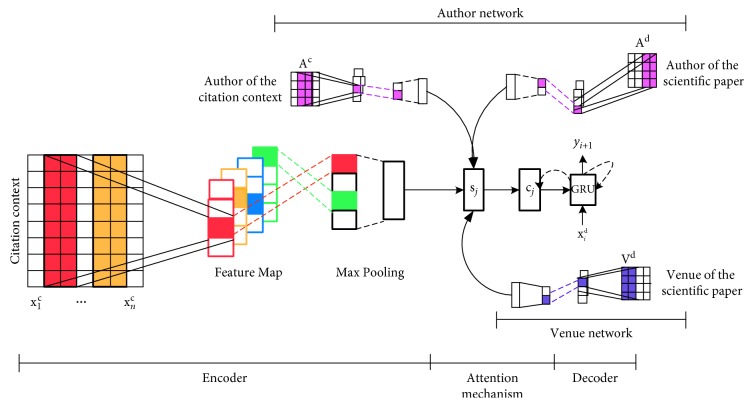
Architecture of the proposed attention-based encoder-decoder (AED) model.

Inspired by the encoder-decoder framework that has been successfully applied in neural machine translation [33, 34], we utilize the encoder-decoder framework in our citation recommendation task. We also combine the attention mechanism in the framework to integrate venue information and author information.  Figure 1 shows the architecture of the proposed AED model.

### 3.1. Encoder

For each given citation context, we use TDNN [35] to obtain convolution on all possible word windows. Then, we use max-pooling nonlinear mapping to extract feature representation for each convolutional word window. Particularly, for a citation context whose length is *l*, we denote the word embedding of the *t*-th word in the citation context as **x**_*t*_^c^ and denote the embedding of the citation context as **x**_1:*l*_^c^=**x**_1_^c^ ⊕ ⋯⊕**x**_*l*_^c^. A convolutional filter **W**^c^ ∈ *R*^*k*·*g*^ (*g* is the dimension of word vector) slides over *k* words at a time over all possible window lengths, i.e., {**x**_1:*k*_^c^, **x**_2:*k*+1_^c^,…, **x**_*l*−*k*+1:*l*_^c^}, and the convolutional layer is defined as

(1) oi=ReLUWTxi:i+k−1+bi,o^=maxo1,…,ol−k+1,

where ReLU is the activation function, **o**_*i*_ is the *i*-th feature map, and **o** ∈ *ℜ*^*l*−*k*+1^. We apply max-pooling operation to obtain the largest feature value extracted by each filter and repeat *p* times yielding ${\hat{\text{o}}}_{j}\in {ℜ}^{p}$. The aim of TDNN is to represent the citation context **X**^c^ as a low-dimensional vector representation **s**_*j*_, i.e.,

(2) $$\begin{array}{c} s_{j}=\tanh\left(U_{s_{j}}{\hat{\text{o}}}_{j}+b_{s_{j}}\right). \end{array}$$

Finally, *f*(**X**^c^) gets different granularity of phrases (i.e., bigrams and trigrams) by using a variety of filters *L*={*l*_1_,…, *l*_|*L*|_}. The phrase level representation captured by the TDNN can improve the performance of semantic representation and meanwhile reduce computational time.

### 3.2. Attention Mechanism

The authors and the venue of a scientific paper have essential impact on the scientific paper. Usually, researchers will track other researchers or research groups which have similar research interests with them. Similarly, the venue of the scientific paper also plays a vital role in whether the scientific paper should be cited, and the authoritative venues are far better than general venues. Therefore, we construct an attention mechanism based on the citation context's authors, the papers' authors, and the papers' venue. We concatenate the vector representations of citation context, citation context's author, the paper's authors, and the paper's venue, yielding

(3) $$\begin{array}{c} s_{j}={\left[f\left(X^{\text{c}}\right)⊕f\left(A^{\text{c}}\right)⊕f\left(A^{\text{d}}\right)⊕f\left(V^{\text{d}}\right)\right]}_{j}, \end{array}$$

where **A**^c^, **A**^d^, and **V**^d^ denote the vector representation of the authors of citation context, the vector representation of the scientific paper's author, and the vector representation of scientific paper's venue. The interaction of citation context, authors, and venues is occurred in decoding process.

### 3.3. Decoder

Since RNN has ability to learn current word from the previous words in the sequence and it can consider the encoder's representation and its internal state at the same time, we use RNN as the decoder. We denote **x**_*i*_^d^ as the word embedding of the *i*-th word in the scientific papers and apply gated recurrent unit (GRU) [36] to solve gradient exploding or vanishing problem. The attention mechanism leans a weighted interpolation **c**_*i*_ based on the encoder's representation, i.e.,

(4) $$\begin{array}{c} c_{i}=\underset{j}{\sum }\alpha_{ij}s_{j}, \end{array}$$

where *α*_*ij*_ is the output value derived from the softmax function and the *i*-th word must be aligned with the *j*-th output.

## 4. Attention-Based Encoder-Decoder Model for Local Citation Recommendation Approach

We map the output of the RNN decoder to the softmax layer, obtaining

(5) $$\begin{array}{c} P\left(\left.y_{i}\right|y_{\leq i},s\right)=\text{softmax}\left(Vh_{i}\right), \end{array}$$

where *P*(*y*_*i*_|*y*_≤*i*_, **s**) is the conditional probability of all previous words in the scientific papers prior to the *i*-th word. We simultaneously train the encoder-decoder using stochastic gradient descent (SGD) [37] and maximize the following equation:

(6) $$\begin{array}{c} \log\;P\left(\left.y\right|X^{\text{c}},X^{\text{d}},A^{\text{c}},A^{\text{d}},V^{\text{d}}\right)=\overset{m}{\underset{i}{\sum }}\log\;P\left(\left.y_{i}\right|y_{\leq i},s\right), \end{array}$$

where **X**^d^ is the scientific papers' vector representation and *m* is the word number of the scientific paper. Once the network is fully trained, the scientific papers *y* can be scored based on the citation context's vector representations **X**^c^, author information **A**^c^ and **A**^d^, and venue information **V**^d^ via equation (6). Those papers which have higher raking score values are selected to generate a recommendation list.

## 5. Experiments

### 5.1. Datasets

We use AAN (http://tangra.cs.yale.edu/newaan/) and DBLP (https://dblp.uni-trier.de/) datasets to estimate the AED model-based approach's performance. As DBLP dataset contains about 1 million bibliography records from the DBLP web set, we select a subset of it, i.e., computer security (ISI, NDSS, ARES, ACSAC FC, and SP), information retrieval (AIRS, CIKM, SIGIR, JCDL, ICTIR, ECIR, TREC, and WSDM), computer vision (ECCV, MM, CVPR, ICPR, ICIP, ACCV, and ICCV), networks and communications (MOBICOM, INFOCOM, ICNP, SECON, ICC, ICDCS, GLOBECOM, and SIGCOMM), and machine learning (ICDE, PAKDD, ICDM, SIGKDD, ICML, NIPS, and WSDM).

Those papers which have no abstracts or titles are removed from the datasets, and the remaining papers are used for experiments. We divide DBLP dataset as a test set and a training set; the training set contains 57,162 papers which are published before 2013 (included), and the test set contains 8,657 papers which are published in 2014 and 2015. AAN dataset is divided in the same way, and the training set of AAN contains 11,197 papers published from 1965 to 2013; the test set contains 1,358 papers. We choose the preceding three sentences and succeeding three sentences of each citation placeholder in each paper, and they constitute the local citation context. Besides, we extract each paper's abstract and title as its text content.

### 5.2. Evaluation Index

We use the following three evaluation indices to evaluate the local citation recommendation's performance.

#### 5.2.1. Recall@*N*

It is defined as the ratio of ground-truth papers appearing in top *N* papers of the recommended list. For this, we evaluate the recall index through *N* = 30, 70, 100.

#### 5.2.2. Mean Average Precision (MAP)

It measures average precision reflecting the rank position regarding the retrieval recommendation list. This indicator is based on the position of the corresponding label values for the top *K* recommended reference papers. We measure the indicator with *K* = 40.

#### 5.2.3. Normalized Discounted Cumulative Gain (NDCG)

It measures the performance of a recommender system based on the graded relevance of the recommended papers. We calculate NDCG value using the following formula:

(7) $$\begin{array}{c} \text{NDCG}@n=Z_{n}\overset{n}{\underset{j=1}{\sum }}\frac{2^{R\left(j\right)}-1}{\log\left(1+j\right)}, \end{array}$$

where *R*(*j*) represents the *i*-th paper's rating value in the ranking list. If the retrieved paper is relevant, then *R*(*j*)=1; otherwise *R*(*j*)=0. *n* is the position and *Z*_*n*_ is a normalization factor.

### 5.3. Experiments and Discussion

For the proposed AED model, we set the dimension of embeddings, memory cell sizes, batch sizes, and feature maps to 128.

#### 5.3.1. Performance of the AED-Based Citation Recommendation Approach

First, we aim to test whether other information beyond content information can enhance the citation recommendation's performance, so we use scientific papers' content information only. We run four sets of experiments: (1) AED-C, which incorporates content information of scientific papers, (2) AED-CA, which incorporates author and content information of scientific papers, (3) AED-CV, which incorporates content and venue information of scientific papers, and (4) AED-CAV, which incorporates scientific papers' author, venue, and content information.  Table 1 shows the results of the above methods.

As scientific papers' content information can only provide coarse information, it leads to low similarity between the two text objects, i.e., scientific papers and citation context; thus, the AED-C approach performs poorest. Experimental results show the performance of AED-CV is inferior to that of AED-CA. We deem the reason is that the author information can provide fine-grained information than the venue information. We found that when we fully utilize scientific papers' author, venue, and content information, we can obtain more accurate reference papers based on the given citation context.

Besides, we investigate whether personal information based on citation context can result in more relevant and individualized references. We denote the personalized citation context as *c*_1_=[*c*_t_, *c*_a_] and denote the non-personalized citation context as *c*_2_=[*c*_t_]. When an academic newcomer, who does not have any publication, submits a citation context, the personalized AED based approach automatically reduces to non-personalized AED-based approach, since the citation context contains only content information *c*_t_.  Table 2 illustrates the experimental results.

#### 5.3.2. Comparison with Other Baseline Approaches

To validate the efficiency of the proposed AED-based approach, we compare it with the following approaches: (1) Context-Aware Relevance (CAR) model [24], which is a nonparametric probabilistic model to recommend references for a given text segment; (2) Neural Citation Network (NCN) model [11], which integrates author information to enhance the performance local citation recommendation performance, and (3) Translation Retrieval (TR) model [31], which narrows the semantic gap between the paper's content and the citation context. Experimental results are listed in Table 3.

From the table, we found that CAR-based approach performs poorest; this can be due to the CAR approach using vector representation based on bag-of-words that loses the valuable semantic information between scientific papers and the given citation contexts. TR approach is better than CAR approach as the TR approach evaluates the semantic relevance between scientific papers and the given citation contexts. However, both CAR approach and TR approach perform worse than NCN approach. This is because CAR approach and TR approach concentrate on scientific papers' content information only, but NCN approach utilizes author information besides content information. We found that the AED approach performs best in these four approaches. We attribute it to that the AED approach simultaneously utilizes multiple information related of scientific papers.

## 6. Conclusion

We develop an attention-based encoder-decoder (AED) model for local citation recommendation in this paper; it first constructs an encoder to represent a citation context as a vector in a low-dimensional space, then it constructs an attention mechanism integrating venue information and author information, and uses RNN to construct a decoder; finally, it maps the decoder's output into a softmax layer and scores the scientific papers via the given citation context, author information, and venue information. A recommended reference paper list is generated based on scientific papers which have high score values. We conduct experiments on the DBLP and ACL Anthology Network (AAN) datasets, and the results illustrate that the performance of AED based approach is better than the other three baseline approaches.

## Figures and Tables

**Table 1:** Experimental results of different AED model-based approaches.

Dataset	Approach	MAP	Recall@30	Recall@70	Recall@100	NDCG@30	NDCG@70	NDCG@100
AAN	AED-C	0.279	0.593	0.656	0.728	0.628	0.716	0.745
	AED-CV	0.288	0.612	0.663	0.740	0.633	0.735	0.748
	AED-CA	0.291	0.618	0.670	0.743	0.639	0.741	0.755
	AED-CAV	0.301	0.625	0.692	0.755	0.652	0.766	0.780


DBLP	AED-C	0.275	0.563	0.641	0.683	0.597	0.695	0.724
	AED-CV	0.289	0.576	0.663	0.705	0.605	0.716	0.735
	AED-CA	0.292	0.578	0.667	0.711	0.616	0.723	0.744
	AED-CAV	0.305	0.591	0.684	0.732	0.627	0.735	0.758

**Table 2:** Comparison of performance between personalzied and non-personalzied approaches.

Dataset	Approach	MAP	Recall@30	Recall@70	Recall@100	NDCG@30	NDCG@70	NDCG@100
AAN	AED-CAV, *c*_1_	0.305	0.632	0.707	0.764	0.663	0.775	0.798
	AED-CAV, *c*_2_	0.301	0.625	0.692	0.755	0.652	0.766	0.780


DBLP	AED-CAV, *c*_1_	0.306	0.597	0.695	0.746	0.633	0.746	0.767
	AED-CAV, *c*_2_	0.305	0.591	0.684	0.732	0.627	0.735	0.758

**Table 3:** Comparison of different local citation recommendation approaches.

Dataset	Approach	MAP	Recall@30	Recall@70	Recall@100	NDCG@30	NDCG@70	NDCG@100
AAN	AED-CAV, *c*_2_	0.301	0.625	0.692	0.755	0.652	0.766	0.780
	NCN	0.282	0.597	0.671	0.726	0.640	0.751	0.772
	TR	0.261	0.578	0.653	0.703	0.629	0.745	0.766
	CAR	0.253	0.566	0.644	0.693	0.618	0.732	0.757


DBLP	AED-CAV, *c*_2_	0.305	0.591	0.684	0.732	0.627	0.735	0.758
	NCN	0.280	0.575	0.653	0.718	0.617	0.724	0.748
	TR	0.262	0.559	0.635	0.686	0.609	0.718	0.735
	CAR	0.245	0.544	0.629	0.683	0.601	0.705	0.730

## Data Availability

The data used in our manuscript can be downloaded from the following two URLs: http://tangra.cs.yale.edu/newaan/ and https://dblp.uni-trier.de/. The two URLs have been also presented in the manuscript (refer Section 5.1).
